# Powerful Tribocatalytic Degradation of Methyl Orange Solutions with Concentrations as High as 100 mg/L by BaTiO_3_ Nanoparticles

**DOI:** 10.3390/nano15141135

**Published:** 2025-07-21

**Authors:** Mingzhang Zhu, Zeren Zhou, Yanhong Gu, Lina Bing, Yuqin Xie, Zhenjiang Shen, Wanping Chen

**Affiliations:** 1 College of Physics and Electronic Engineering, Hainan Normal University, Haikou 571158, China; 202412070200020@hainnu.edu.cn (M.Z.); 410901@hainnu.edu.cn (L.B.); 202312070200012@hainnu.edu.cn (Y.X.); 2 School of Physics and Technology, Wuhan University, Wuhan 430072, China; 2019302020164@whu.edu.cn; 3 School of Physics and Electronic Information, Key Lab Electromagnet Transformat & Detect Henan, Luoyang Normal College, Luoyang 471022, China; guyanhong@lynu.edu.cn

**Keywords:** tribocatalysis, degradation, methyl orange, BaTiO_3_ nanoparticles, coatings

## Abstract

In sharp contrast to photocatalysis and other prevalent catalytic technologies, tribocatalysis has emerged as a promising technology to degrade high-concentration organic dyes in recent years. In this study, BaTiO_3_ (BTO) nanoparticles were challenged to degrade methyl orange (MO) solutions with unprecedentedly high concentrations through magnetic stirring. With BTO nanoparticles and home-made PTFE magnetic rotary disks in 50 mg/L MO solutions, 10 h of magnetic stirring resulted in 91.4% and 98.1% degradations in an as-received glass beaker and in a beaker with a PTFE disk coated on its bottom, respectively. Even for 100 mg/L MO solutions, nearly complete degradation was achieved by magnetic-stirring-stimulated BTO nanoparticles in beakers with the following four kinds of bottom: 97.3% degradation in 30 h for a glass bottom, 97.4% degradation in 20 h for a PTFE coating, 97.9% degradation in 42 h for a Ti coating, and 97.4% degradation in 74 h for an Al_2_O_3_ coating. Electron paramagnetic resonance (EPR) analyses revealed that the generation of reactive oxygen species (ROS) by magnetic-stirring-stimulated BTO nanoparticles is dramatically affected by the bottom material of beakers. These findings suggest an appealing prospect for BTO nanoparticles to utilize mechanical energy for sustainable water remediation.

## 1. Introduction

Global environmental protection has become increasingly important, and water resource contamination and remediation have attracted significant attention [1,2]. For instance, over 10,000 tons of synthetic dyes are used in textile industries every year, which usually do not bind tightly to fabrics and partially enter into wastewater, posing serious ecotoxicological threats to various living organisms [3]. Conventional wastewater treatment technologies, such as adsorption, membrane filtration, and coagulation–flocculation, are generally plagued by high costs, low efficiency, and secondary pollution risks, failing to fully meet current environmental protection demands [4,5,6]. Consequently, the development of novel, efficient, and green technologies is urgently required.

In this context, it is highly desirable to utilize more clean energies in nature for environmental remediation. Photocatalysis is an appealing green technology for this purpose. Solar light is absorbed by semiconductor nanoparticles suspended in water, through which electron-hole pairs are excited in semiconductor nanoparticles and initiate various redox reactions in ambient water [7,8,9,10,11]. However, the absorption of solar light by semiconductor nanoparticles is adversely affected by the low transparency of wastewater [12,13]. As the transparency of organic-dye-containing wastewater decreases with an increase in organic dye concentration, it is a significant challenge for photocatalysis to treat the wastewater of high-concentration organic dyes. Mechanical energy is another form of clean energy abundant in nature, and in recent years tribocatalysis has emerged as a research hotspot for utilizing mechanical energy for wastewater treatment [14,15,16,17]. This technology generates reactive oxygen species (ROS, such as •OH and $•O_{2}^{-}$) on catalyst surfaces through friction, which is not directly affected by the transparency of wastewater. So, in principle, tribocatalysis has the potential to be developed for the treatment of low-transparency wastewater [18]. Although the tribocatalytic performance of some materials was found to dramatically decrease with an increase in dye concentration [19,20], tribocatalysis has been reported to treat organic dyes with increasing concentrations, suggesting a broader scope for its application in environmental remediation [21,22,23,24,25].

The chemical stability of organic dyes differs greatly from one another. Among the common organic dyes, RhB dye is relatively easy to degrade due to the prominent presence of low-energy bonds (C–C and C–N) in its molecules, while MO is much more difficult to degrade than other common organic dyes because of the presence of high-energy bonds (C=N and N=N) in its molecules [26,27]. To date, tribocatalysis has been successfully applied to degrade 50 mg/L RhB and 30 mg/L MB, while only 20 mg/L MO [28,29,30]. Tribocatalysis had been applied to degrade 30 mg/L MO, in which MO molecules were only broken into small organic chemicals like benzoic acid, succinic acid, and p-phenol [25]. Roughly speaking, the tribocatalytic degradation of MO solutions with higher concentrations represents a significant challenge in environmental remediation.

With exceptional stability and ferroelectric properties, BaTiO_3_ (BTO) nanoparticles have been extensively investigated for applications in various forms of catalysis [31,32,33,34,35,36]. In a recent study, BTO nanoparticles were successfully applied as a tribocatalyst to degrade 20 mg/L MO [28]. It is well known that for organic dyes, the higher the concentration, the more challenging the degradation. In this study, we challenged BTO nanoparticles to degrade MO solutions with much higher concentrations through magnetic stirring. To our great surprise, MO solutions up to 100 mg/L were effectively degraded by BTO nanoparticles in beakers with four types of bottoms. In particular, for a beaker with a PTFE disk coated on its bottom, 97.4% degradation was achieved after 20 h of magnetic stirring. To the best of our knowledge, there has been no report on the catalytic degradation of such a high-concentration MO solution in the literature. Given the low cost and high stability of both BTO nanoparticles and PTFE, the results obtained in this study suggest a promising potential for tribocatalysis in large-scale environmental remediation.

## 2. Materials and Methods

### 2.1. Materials and Their Characterization

High-purity (99.99 wt%) BTO nanoparticles, purchased from Shanghai Aladdin Biochemical Technology Co., Ltd., Shanghai, China, were used in this study. The crystalline structure of the nanoparticles was characterized by X-ray diffraction (XRD; Philips X-ray diffractometer system, Philips, Amsterdam, The Netherlands) using Cu K radiation. A morphological analysis was performed using a field-emission scanning electron microscope (FESEM; JEOL JSM-6700F, JEOL Ltd., Tokyo, Japan). The particle size distribution (average, maximum, and minimum diameters) was determined via Nano Measure 1.2 software by statistically analyzing marked particles in SEM images.

### 2.2. Formation of Coatings on the Bottoms of Glass Beakers

For the tribocatalytic experiments, commercial flat-bottomed glass beakers (φ 45 mm × 60 mm) were categorized into four groups. The first group was unmodified, with as-received glass bottoms. The other three groups were modified by attaching disks (φ 40 mm × 1 mm) of Ti, Al_2_O_3_ ceramics, and PTFE separately to the bottoms of the beakers using a strong adhesive (deli super glue 502). Consequently, four distinct types of beakers were obtained with bottoms of glass, Al_2_O_3_, Ti, and PTFE.

### 2.3. MO Degradation Tests

In a typical experiment, 0.30 g of BTO nanoparticles was added to a beaker containing 30 mL of 50 or 100 mg/L MO solutions. A home-made PTFE magnetic rotary disk was placed in the suspension and was driven to rotate at 400 rpm in the dark at room temperature (25 °C).

**Figure 1:**
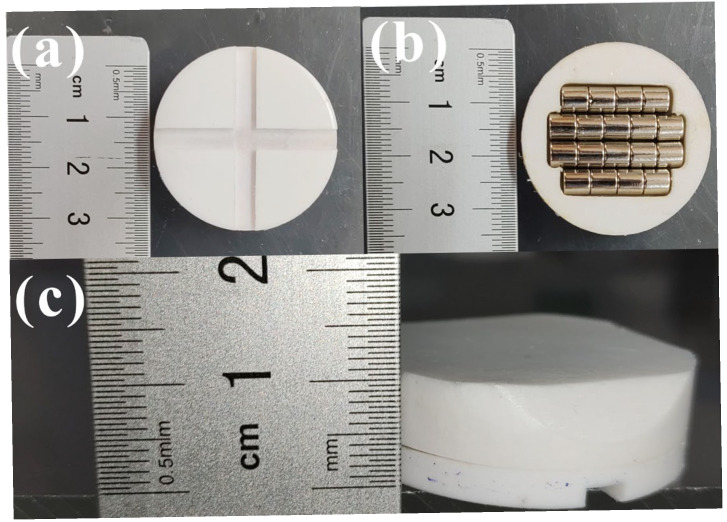
Photographs of home-made PTFE magnetic rotary disk: (**a**) front surface of cross-shaped grooves; (**b**) front surface of top cover; (**c**) side surface.

As depicted in Figure 1, cross-shaped grooves, 4 mm wide and 2 mm deep, were formed on one major surface of Teflon disks 35 mm in diameter and 5 mm in thickness. In addition, we designed a 7 mm thick Teflon top cover. A groove was made in the center of one side of the top cover, and four sets of commercial cylindrical magnets were precisely embedded. For one such Teflon disk, the four groups of commercial cylindrical magnets comprised two that were ф 5 mm × 25 mm and two that were ф 5 mm × 20 mm. Then, we adhered the two surfaces together with 502 glue and sealed the magnet inside. The magnets were mounted to drive the disk to rotate on an ordinary magnetic stirrer. The groove in the disk enabled the particles to enter the interface between the Teflon disk and vessel bottom in the course of magnetic stirring. These Teflon disks with magnets were designed especially for applications in tribocatalysis, and are hereafter termed as PTFE magnetic rotary disks.

At designated intervals, 3 mL aliquots were collected, centrifuged at 8000 rpm for 5 min to remove the nanoparticles, and analyzed using a UV–Vis spectrometer (UV-2550; Shimadzu, Kyoto, Japan) over 200–650 nm. For UV–Vis absorption spectra, the following parameters were set: scan range = 200–650 nm, wavelength interval = 1.0 nm, and slit width = 2 nm. The efficiency of organic dye degradation is typically calculated using the equation D = 1 − A/A_0_, with A_0_ and A denoting the initial and remaining absorbance at the dye’s characteristic peak, respectively.

### 2.4. Detection of Active Species

For the detection of hydroxyl radicals, 10 mL deionized water, 50 µL 5, 5-dimethyl-1-pyrroline-N-oxide (DMPO), and 0.15 g BTO nanoparticles were introduced into four glass beakers (φ 45 mm × 60 mm) with either a glass bottom, an Al_2_O_3_ coating, a Ti coating, or a PTFE coating. For superoxide radical detection, the same setup was employed but with 10 mL methanol replacing the deionized water. Each beaker was fitted with a PTFE magnetic rotary disk to stir the mixture at 400 rpm for 15 min in a dark, ambient-temperature environment. Subsequently, hydroxyl and superoxide radicals were identified using an electron paramagnetic resonance (EPR) spectrometer (A300-10/12; Bruker, Berlin, Germany).

## 3. Results

### 3.1. Material Information

**Figure 2:**
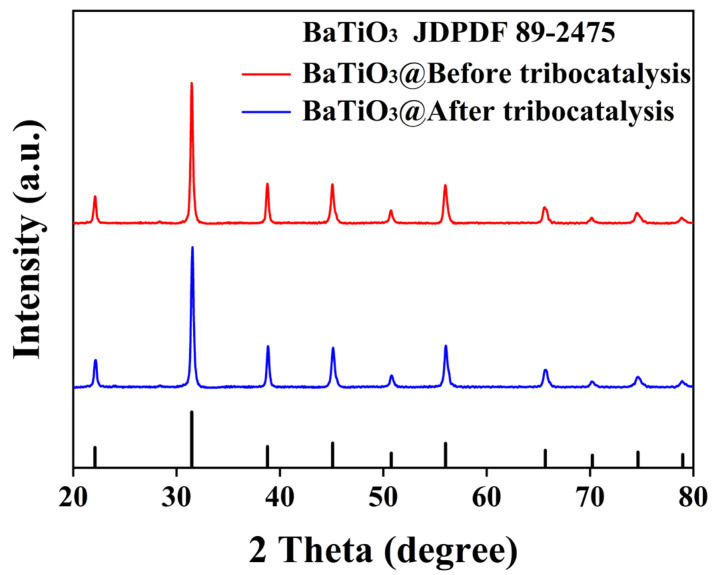
XRD patterns of BTO nanoparticles before and after tribocatalysis.

Figure 2 presents an XRD pattern of the BTO nanoparticles before and after tribocatalysis. All diffraction peaks are from the standard cubic perovskite structure and have been indexed according to the PDF #89-2475 reference card. No impurity peaks are observed, confirming the high purity of the sample. It is clear that the lattice structure of BTO nanoparticles remains unchanged after tribocatalysis. These main peaks are quite strong and sharp, suggesting the high crystallinity of the BTO nanoparticles, which is important for efficient tribocatalysis [16].

**Figure 3:**
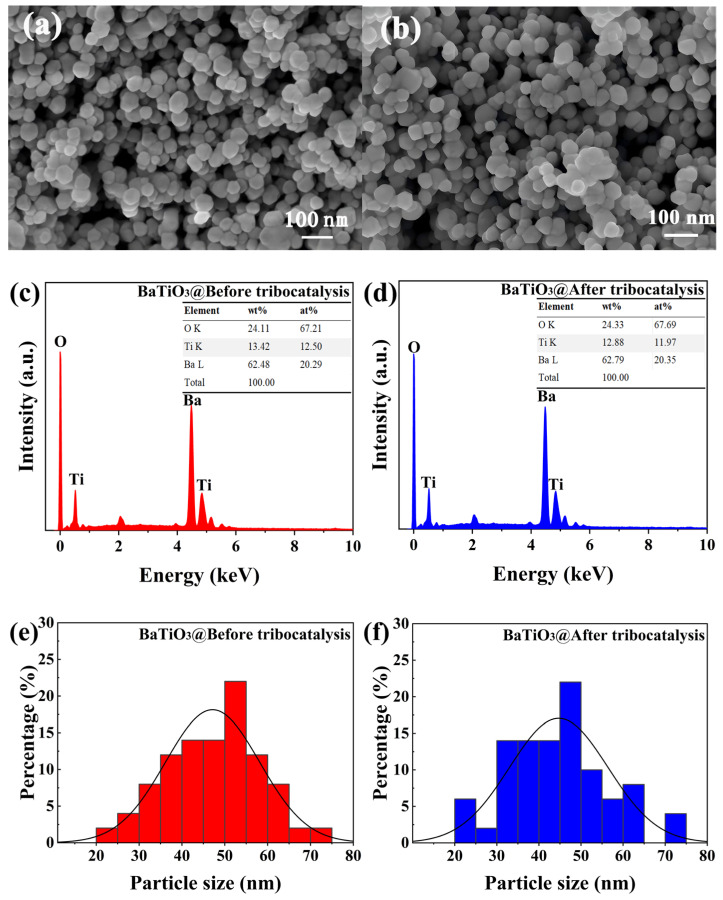
SEM images, EDX spectra, and particle size distributions of BTO nanoparticles: (**a**) SEM before and (**b**) SEM after tribocatalysis; (**c**) EDX before and (**d**) EDX after tribocatalysis; (**e**) particle size before and (**f**) particle size after tribocatalysis.

Figure 3a,b present representative scanning electron microscope (SEM) images of BTO nanoparticles before and after tribocatalysis. Figure 3c,d display the energy-dispersive X-ray spectroscopy (EDX) images of the BTO nanoparticles in the same states. Figure 3e,f show the particle size of the BTO nanoparticles before and after tribocatalysis. The nanoparticles are spherical in shape, with all major elements detected. They have an average diameter of around 50 nm and a size distribution of approximately 20–70 nm. Such a rather spherical morphology is helpful to exclude any potential piezoelectric contributions through deformation of the BTO nanoparticles under magnetic stirring. There are no significant changes in the morphology, elemental composition, or average diameters of BTO nanoparticles after tribocatalysis.

### 3.2. Tribocatalytic Degradation of MO Solutions

**Figure 4:**
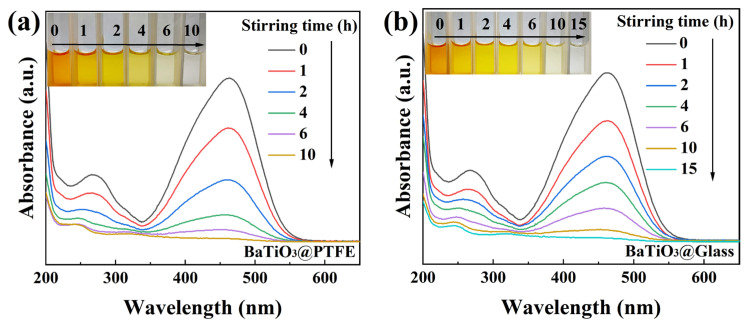
UV–Vis absorption spectra of MO (50 mg/L) solutions mediated by BTO nanoparticles rubbing on different materials (inset: color change of solutions): (**a**) PTFE coating; (**b**) glass bottom.

The presence of high-energy bonds (C=N and N=N) in the molecular structure of MO renders its degradation significantly more challenging than other conventional organic dyes [37]. The current literature reports tribocatalytic degradation of 50 mg/L RhB solutions [28], whereas for MO solutions, concentrations above 30 mg/L remain unstudied [25]. In this study, we first challenged BTO nanoparticles to degrade 50 mg/L MO solutions through magnetic stirring. To our great surprise, tribocatalytic degradation was observed for 50 mg/L MO solutions in the two beakers with glass and PTFE bottoms separately, as shown in Figure 4a,b. In both beakers, the intensity of the MO-specific absorption peak at 464 nm systematically decreased with an increase in the stirring duration, and the MO solution finally became colorless. It should be pointed out that for the tribocatalytic degradation of 30 mg/L MO solutions by Si single crystals, although the solutions became colorless rather quickly, a strong peak around 250 nm appeared in the UV–Vis adsorption spectra [25], which suggests that the MO molecules were only broken into small organic molecules like benzoic acid, succinic acid, and p-phenol. The spectral changes across the entire monitored wavelength range observed in this study demonstrate a quite different degradation of MO molecules, in which the MO molecules decomposed into CO_2_ and H_2_O.

**Figure 5:**
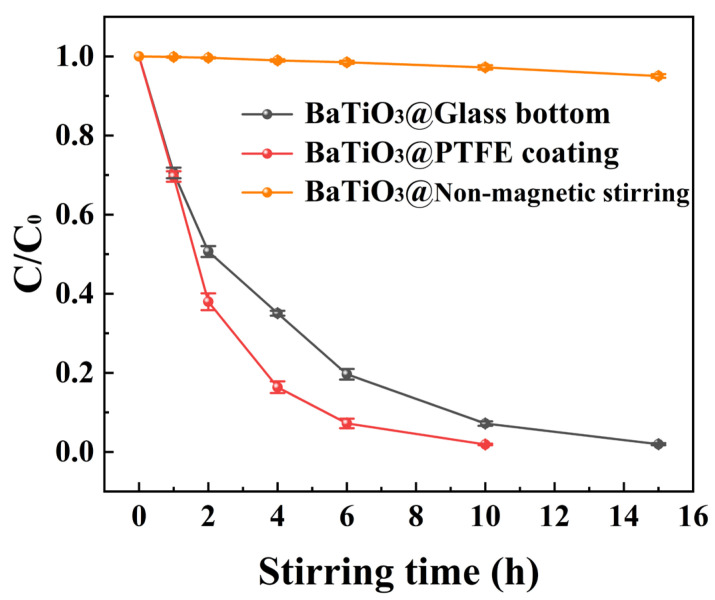
The C/C_0_ ratio versus the duration of magnetic stirring in beakers with either a PTFE coating, a glass bottom, or a glass bottom without magnetic stirring.

Figure 5 compares the degradation curves of MO over time for BTO nanoparticles, comprising multiple experiments conducted in PTFE-coated beakers, glass-bottomed beakers, and glass-bottomed beakers without magnetic stirring. It is evident that with an increase in magnetic stirring time, the C/C_0_ ratio steadily decreased to almost zero in both beakers. So, MO solutions with a concentration up to 50 mg/L were successfully degraded by BTO nanoparticles under magnetic stirring. However, in the absence of magnetic stirring, the degradation of BTO nanoparticles in a glass-bottomed beaker was almost nonexistent.

To further assess the stability of BTO nanoparticles in dye degradation, cycling tests were performed in PTFE-coated and glass-bottomed beakers. In each cycle, 30 mL of a 50 mg/L MO solution suspended with 0.30 g BTO nanoparticles was magnetically stirred for 10 h in a PTFE-coated beaker and 15 h in a glass beaker using a home-made Teflon magnetic rotary disk. Post-stirring, the nanoparticles were separated via centrifugation and the supernatant was measured using a UV–Vis spectrometer. The nanoparticles were washed several times with deionized water and then reused in the next cycle. Six consecutive cycles were conducted in both beakers, and the degradation rates obtained for every cycle are shown in Table 1. Surprisingly, the BTO nanoparticles displayed an excellent high-degradation efficiency for all six consecutive cycles in both beakers. These outcomes demonstrate the highly robust cycling stability of BTO nanoparticles in dye degradation applications.

**Figure 6:**
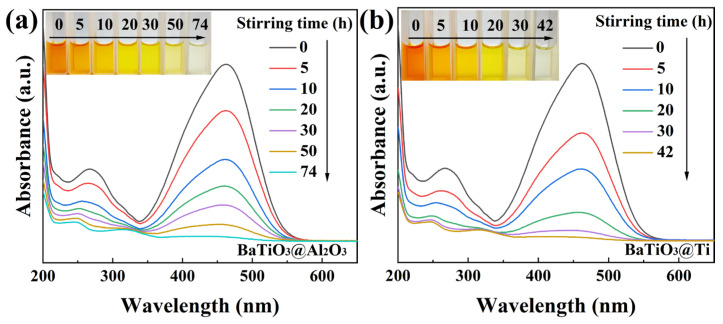

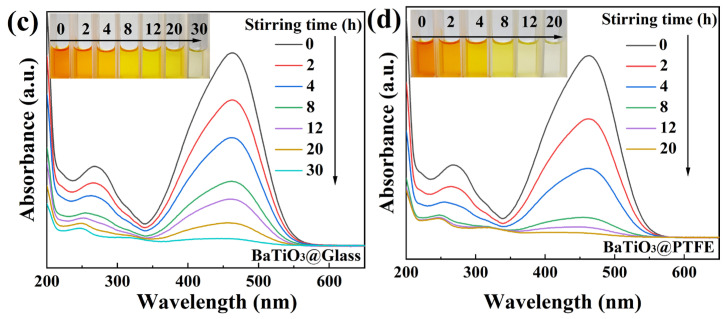
UV–Vis absorption spectra of MO (100 mg/L) solutions mediated by BTO nanoparticles rubbing on different materials (inset: color change of solutions): (**a**) Al_2_O_3_ coating; (**b**) Ti coating; (**c**) glass bottom; (**d**) PTFE coating.

The results obtained for the 50 mg/L MO solutions were so encouraging that we further challenged the BTO nanoparticles to degrade 100 mg/L MO solutions in beakers with four kinds of bottoms, namely, glass, a PTFE coating, a Ti coating, or an Al_2_O_3_ coating. Quite unexpectedly, the 100 mg/L MO solutions degraded rather thoroughly in all the beakers, although the time needed for magnetic stirring dramatically differed from one beaker to another. As shown in Figure 6a, for the 100 mg/L MO solution in the Al_2_O_3_-coated beaker, the degradation rate reached 97.4% and the solution became colorless after 74 h of magnetic stirring. As shown in Figure 6b, for the 100 mg/L MO solution in the Ti-coated beaker, the degradation rate reached 97.9% and the solution became colorless after 42 h of magnetic stirring. In the glass-bottomed beaker containing a 100 mg/L MO solution, the degradation rate was 97.3% following 30 h of magnetic stirring, as depicted in Figure 6c. The most remarkable result was observed for the 100 mg/L MO solution in the PTFE-coated beaker, whose degradation rate reached 97.4% after only 20 h of magnetic stirring, as shown in Figure 6d. As a ferroelectric material with a perovskite structure, BTO has been extensively studied for its versatile catalytic properties. It should be emphasized that the power of the magnetic stirring used in this study was only around 3 W, which is much weaker than the power consumed in most photocatalytic or piezocatalytic investigations of BTO nanoparticles. The degradation of the 100 mg/L MO solutions by BTO nanoparticles in this study thus represents a major advancement in the catalytic properties of BTO nanoparticles.

**Figure 7:**
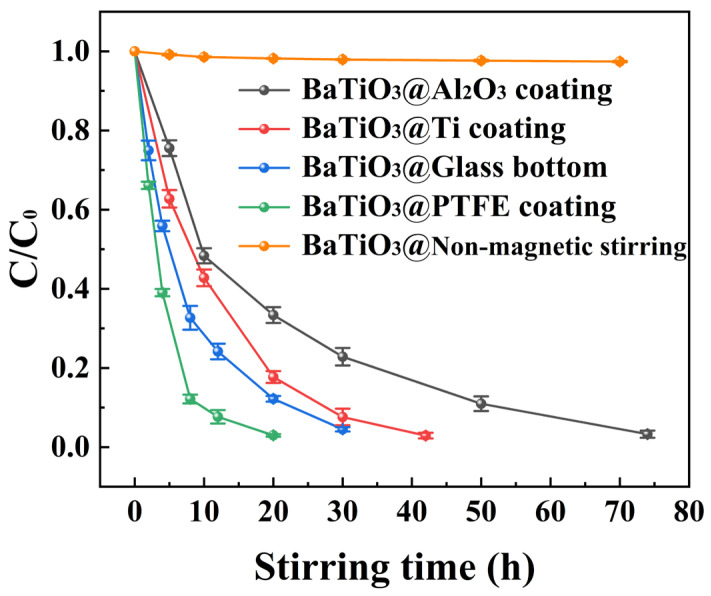
C/C_0_ ratio versus duration of magnetic stirring for tribocatalytic degradation of 100 mg/L MO solutions by BTO nanoparticles in beakers with either an Al_2_O_3_ coating, a Ti coating, a glass bottom, a PTFE coating, or a glass bottom without magnetic stirring.

Figure 7 compares the curves of the C/C_0_ ratio versus the duration of magnetic stirring for the tribocatalytic degradation of 100 mg/L MO solutions by BTO nanoparticles from multiple experiments. These experiments were conducted in beakers with the following different configurations: an Al_2_O_3_ coating, a Ti coating, a glass bottom, a PTFE coating, and a glass bottom without magnetic stirring. It is clear that 100 mg/L MO solutions were steadily degraded by magnetic-stirring-stimulated BTO nanoparticles in all the four beakers with different bottoms. However, the degradation of BTO nanoparticles in glass-bottomed beakers without magnetic stirring was also almost nonexistent. Many investigations have shown that there is a negative correlation between tribocatalytic degradation efficiency and initial dye concentrations [38]. Specifically, for RhB solutions suspended with Bi_12_TiO_20_ nanoparticles, the dye concentration remained nearly constant after 12 h of magnetic stirring when the RhB concentration was raised to 30 mg/L [20]. Given these facts, the results shown in Figure 7 should greatly update our knowledge of tribocatalysis that has been achieved to date.

**Figure 8:**
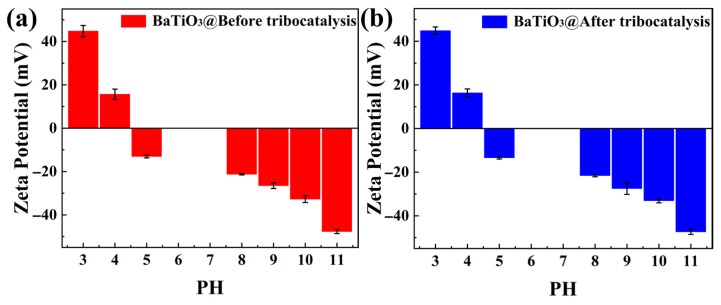
Zeta potentials of BTO nanoparticles: (**a**) before tribocatalysis; (**b**) after tribocatalysis.

Figure 8a shows the variations in the zeta potential with pH for BTO nanoparticles before tribocatalysis. Figure 8b shows the corresponding variations after tribocatalysis. As mentioned in the literature, researchers assess the stability of materials at different pH values based on the absolute magnitude of the zeta potential [39]. When the absolute zeta potential is high, the particles are not prone to aggregation due to electrostatic repulsion. Conversely, when the absolute zeta potential is low, particles tend to aggregate due to forces such as van der Waals interactions, leading to decreased system stability. By comparing Figure 8a,b, we observed few differences between them. Furthermore, the absolute zeta potential values remained relatively high across the tested pH range, both before and after treatment. This indicates that the BTO nanoparticle system possesses good overall stability, enabling it to undergo sufficient tribocatalytic reactions with MO pollutants over multiple cycles.

**Figure 9:**
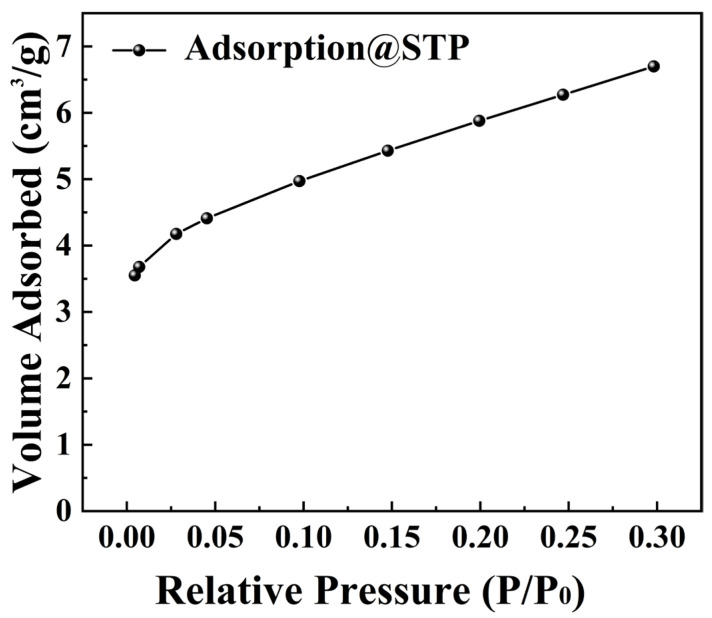
BET surface area data of BTO nanoparticles.

Figure 9 presents the adsorption isotherm of BTO nanoparticles at 200 °C. An analysis using the BET method revealed a surface area of 20.805 m^2^/g. This indicates their relatively low surface area. Our study shows that the low surface area of BTO nanoparticles correlates with a weak adsorption capacity. This implies that the excellent tribocatalytic degradation of organic dyes by BTO nanoparticles is not linked to their adsorption properties.

### 3.3. Mechanism Study on Tribocatalytic Degradation of MO by BaTiO_3_ Nanoparticles

For those materials with a semiconductor-type band gap, a tribocatalysis mechanism has been established in which electron-hole pairs are excited by the mechanical energy absorbed through friction [14]. For the BTO nanoparticles in this study, this mechanism can be expressed as follows:

(1) $$BaTiO_{3}\;NPs\overset{Friction}{\to }BaTiO_{3}\;NPs+h^{+}+e^{-}$$

Concurrently, holes oxidize surface-adsorbed hydroxide ions to produce hydroxyl radicals (•OH), while conduction band electrons participate in redox reactions with molecular oxygen, yielding superoxide radical anions ($•O_{2}^{-}$), as demonstrated in the following reaction sequence:

(2) $${OH}^{-}+h^{+}\to \hat{A}\cdot OH$$

(3) $$O_{2}+e^{-}\to \hat{A}\cdot O_{2}^{-}$$

The generated •OH and $•O_{2}^{-}$ radical species, electrons, and holes initiate cascade oxidative degradation pathways with organic chromophores, ultimately mineralizing complex dye structures into environmentally benign byproducts such as CO_2_, H_2_O, and low-molecular-weight inorganic compounds, as governed by the following reaction kinetics:

(4) $$h^{+}/e^{-}/A\cdot OH/A\cdot O_{2}^{-}+MO\to degradation$$

**Figure 10:**
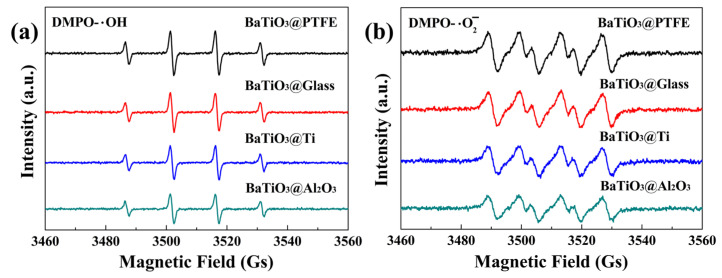
EPR spectra obtained for BTO nanoparticles stirred using PTFE magnetic rotary disks in glass and PTFE—, Ti—, and Al_2_O_3_—coated beakers containing (**a**) deionized water with DMPO as the spin-trapping agent and (**b**) methanol with DMPO as the spin-trapping agent.

An EPR spectroscopy analysis at a microwave frequency of 9.85 GHz and a modulation frequency of 100 kHz revealed radical generation by BTO nanoparticles under magnetic stirring. For the EPR analysis, the following parameters were set: center field = 3510.000 G, sweep width = 100.000 G, static field = 3460.000 G, modulation amplitude = 1.00 G, and resolution of X = 1024. After BTO nanoparticles were stirred in deionized water for 15 min, characteristic four-peak hydroxyl radical signals (intensity ratio of 1:2:2:1) were detected for the glass and PTFE-, Ti-, and Al_2_O_3_-coated beakers (Figure 10a). Parallel experiments in methanol showed four equal-intensity peaks (1:1:1:1 ratio) corresponding with superoxide radicals (Figure 10b). Although these signals of hydroxyl and superoxide radicals were quite different in intensity from one beaker to another, a clear correlation can be observed between the results shown in Figure 10 and those shown in Figure 6. Among the four kinds of glass beakers, the stronger the hydroxyl and superoxide radical signals shown in Figure 10, the better the tribocatalytic performance of the BTO nanoparticles shown in Figure 6. The tribocatalytic degradation process of MO by BTO nanoparticles was, therefore, well-expressed by Equations (1)–(4).

**Figure 11:**
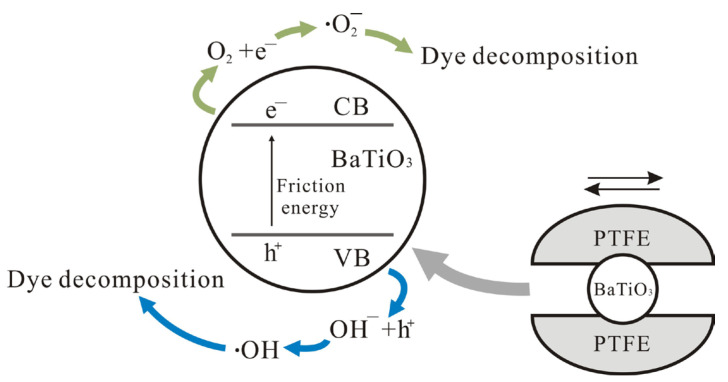
A schematic illustration of tribocatalytic organic dye degradation by BTO nanoparticles in PTFE-coated beakers.

The formation of functional coatings on the bottoms of beakers or reaction vessels has emerged as a simple but effective strategy for enhancing the tribocatalytic degradation of organic pollutants, as well as regulating the transformation of H_2_O and CO_2_ [18]. However, it is highly challenging to understand the effects of these coatings on tribocatalysis [40,41,42,43,44]. As BaTiO_3_ nanoparticles showed the best tribocatalytic performance in PTFE-coated beakers, and as the fewest kinds of materials took part in the tribocatalytic degradation process in this case, we created a schematic diagram to illustrate the corresponding tribocatalysis mechanism, as shown in Figure 11. It can safely be concluded that the friction between BTO and PTFE must be especially effective to excite electron-hole pairs in BTO; consequently, BTO nanoparticles showed the best tribocatalytic performance at degrading MO molecules in PTFE-coated beakers.

Compared with titanium, Al_2_O_3_, and glass coatings, PTFE exhibits lower mechanical hardness. This property enables a greater contact area with BTO nanoparticles during tribocatalysis, thereby enhancing electron exchange efficiency. Our hydroxyl radical (•OH) detection results confirm that PTFE coatings generate significantly more •OH and superoxide radicals ($•O_{2}^{-}$) than Ti, Al_2_O_3_, or glass coatings. As radical generation inherently involves electron transfer processes, these findings demonstrate superior electron exchange efficiency at PTFE interfaces. This performance enhancement originates from PTFE’s unique triboelectric properties, enabling a superior charge transfer, chemical inertness, and a surface morphology facilitating sustained friction interfaces. As reported in the literature [45,46,47,48], enhanced interfacial charge dynamics and the suppressed electron-hole recombination of photogenerated carriers directly correlate with improved catalytic degradation efficiency.

The tribocatalytic degradation of MO has been investigated for many materials, and the results obtained for Si, CdS, and BaTiO_3_ in this study present an interesting contrast. For Si, a peak at around 250 nm in the UV–Vis adsorption spectra always appeared when it created friction with glass and Al_2_O_3_ [25]. For CdS, a peak around 250 nm appeared when it created friction with glass and Ti but did not appear when it rubbed against Al_2_O_3_ [29,30]. For BTO in this study, such a peak was not observed for all four kinds of bottom materials. Among Si, CdS, and BaTiO_3_, Si has the smallest band gap while BaTiO_3_ has the largest band gap [49]. Although a general tribocatalytic mechanism based on the excitation of electron-hole pairs by friction energy has been proposed for semiconductor materials, the contrast revealed here suggests that the tribocatalytic dye degradation pathway created by semiconductor materials shows dependence on some specific factors. It will be highly meaningful to further explore the influence of the band structure on the tribocatalytic performance of semiconductor materials.

## 4. Conclusions

Stimulated through magnetic stirring with PTFE magnetic rotary disks, BTO nanoparticles achieved a rather high tribocatalytic degradation rate of high-concentration MO solutions in different circumstances, including 98.3% for 50 mg/L MO in 15 h and 97.3% for 100 mg/L MO in 30 h for glass-bottomed beakers, 98.1% for 50 mg/L MO in 10 h and 97.4% for 100 mg/L MO in 20 h for PTFE-coated beakers, 97.9% for 100 mg/L MO in 42 h for a Ti-coated beaker, and 97.4% for 100 mg/L MO in 74 h for an Al_2_O_3_-coated beaker. An electron paramagnetic resonance (EPR) analysis confirmed the influence of beaker-bottom materials on the generation of reactive oxygen species (•OH and $•O_{2}^{-}$) by magnetic-stirring-stimulated BTO nanoparticles. Despite the dramatic variation in the time needed to achieve a high tribocatalytic degradation rate for high-concentration MO solutions in different circumstances, this work reveals a promising prospect for BTO nanoparticles to utilize mechanical energy to treat wastewater with high-concentration organic dyes.

## Figures and Tables

**Table 1:** Cycling stability of BTO nanoparticles: tribocatalytic degradation rate of 50 mg/L MO solution after 10 h and 15 h of magnetic stirring in PTFE-coated and glass-bottomed beakers, respectively.

	Cycle Number					
	1	2	3	4	5	6
Degradation rate @PTFE	0.988	0.958	0.969	0.959	0.952	0.959
Degradation rate @glass	0.979	0.984	0.969	0.990	0.986	0.986

## Data Availability

The original contributions presented in the study are included in the article; further inquiries can be directed to the corresponding authors.
